# The provenance of trans/gender: on the subject’s willful disappearance from the record

**DOI:** 10.1007/s10502-025-09477-x

**Published:** 2025-02-14

**Authors:** L. Wynholds

**Affiliations:** University of Utah, Salt Lake City, UT USA

**Keywords:** Information structures, Documentation of gender, Provenance of gender records, Health records, Data ethics, Hostile recordkeeping, Recordkeeping practices, Situational analysis, First person authority, Standards of care, Marginalized populations, Stigma, Gender identity, SOGI surveys, Unsafe spaces, Sensitive health data, Health Information Exchange, Gatekeeping, History of medicine, Administrative violence, Medical records, Health data, LGBTQ + health care, Denial of care, Participatory archiving, Archival autonomy, Willful indifference, Medical care, Transgender health, Stigmatizing language in health care, Unconscious bias, Implicit bias

## Abstract

In recent years, health and government information systems have made gender variance more visible and countable in the records. However, being counted has created novel forms of vulnerability for transgender and gender-diverse populations. This paper explores the complexity of records in exposing vulnerabilities of marginalization, illustrating how recordkeeping practices may contribute to erasure and stigma and reproduce systems of social disenfranchisement. The paper introduces the concept of ‘hostile recordkeeping practices’ as a critical analytical lens to articulate observations of marginalization in recordkeeping environments, to discuss indicators of bias, and to develop mechanisms for surfacing issues of trauma, violence, stigma, and repair. Using examples from the published literature, the paper discusses the importance of understanding social interactions which exacerbate marginalization within recordkeeping practices. Based on a case study, the paper argues that the dangers trans and gender-diverse individuals routinely encounter in recordkeeping practices necessitate additional analytical inquiry into hostile recordkeeping practices. The case study contributes to discussions of ethics of care for records for marginalized populations and includes interactional considerations, ethical considerations and historical contexts embedded in recordkeeping systems. The paper closes with an argument for the need to adapt existing recordkeeping practices to safeguard against hostile uses, systemic bias and structural violence. Future archival and medical discourses alike need to engage with the situation(s) of creation, the acts of recording, and, most importantly, personal agency in the negotiations of allowable uses for the records for marginalized populations.

## Introduction

2020 was a period in the United States of shifting mindsets, culminating in a landslide of social and political changes. The presidential election polarized people across the nation and the globe. A novel respiratory virus (COVID-19) evolved into a worldwide pandemic and exposed deep social tensions around public health. George Floyd and Black Lives Matter protests became lightning rods for political conflict. During these events, public hostility toward government records demonstrated a profound loss of faith in benign outcomes, especially in relation to the practice of medicine. At the same time, I also experienced a profound loss of faith. Mine was around the ethics of recordkeeping for marginalized populations. When I started working with health records for trans and gender-diverse populations (referred to hereafter as trans+), it was widely assumed that being included in recordkeeping systems (e.g., in the records, software applications, data processes, and professional activities) was beneficial and would reduce negative health outcomes (Wynholds 2018). However, after more than a decade in the field, I had a series of experiences which foundationally changed how I viewed records for marginalized populations and changed my understanding of recordkeeping as a practice. Caroline Paul (2013), in her autobiographical novel, ‘Lost Cat,’ articulated similar feelings after a life-changing event:

“In the blink of a left wingtip I had lost the delusion that the universe was benign and that we were at the center of its doting love. In short, I had realized that not everything works out just fine. Things can go to hell fast, and never return to normal.”* (p. 11)*

At the center of my change were deep questions about how institutions create, deposit, and give access to records, particularly in relation to the burdens of social biases and hostility for marginalized populations. Up until that point, I had generally seen government and medical records as benign and largely objective reflections of institutionally structured interactions. Afterward, I became highly critical of the lack of safeguards in place to mitigate bias, reduce harms and document hostility articulated toward marginalized communities or individuals. This paper argues for more critical inquiries into the lack of safeguards in recordkeeping practices and serves as a starting point for practitioners to incorporate these concerns into larger archival discourses.

Marginalized populations often have complex and problematic relationships with medical and state recordkeeping paradigms, which include histories of denials of personal agency, community organizing, histories of trauma, histories of erasure, and experiences of stigma. Building from these contexts, this paper argues for the addition of a foundational concept of a hostile recordkeeping environment for archival studies, asserting that the concept is essential to the understanding of provenance for records documenting marginalized populations. The paper builds the argument for the need for the concept of hostile recordkeeping via:

I) Introducing the concept of hostile recordkeeping practices,
II) Introducing related concepts from archival theory, gender theory, and the social construction of records,
III) Grounding the need for the concept from population-based observational studies,
IV) Providing a case study to illustrate the complexity of hostile recordkeeping practices,
V) Reflecting on the records and their intersections with hostile recordkeeping practices, and
VI) Concluding with a call for archival professionals to work to address the ethical questions surfaced by records produced within hostile recordkeeping practices.

The paper uses a sample case study to illustrate the immense complexity of records situated within marginalized populations, histories of trauma, agents of unconscious bias, violence and cultural hostility. Throughout the paper, I contrast cultural assumptions of safety with evidence from the literature documenting active harms. The paper closes with a discussion of the complexity of the role of records in transformative justice and the question of what happens if official records cannot be safeguarded from institutional harms, bias, violence or cultural hostility.

## Introducing the concept of hostile recordkeeping

Looking back through various historical periods, there are dramatic examples of government records being used to target and harm persons within the records. During the German occupation of the Netherlands in the 1940s, population records were collected from local government offices. These records were used in subsequent projects to eliminate population groups which were classified as ‘undesirable.’ These actions were still part of the present day for many survivors and their descendants. Stories about family records from WWII are still being actively circulated:

“The original documents were burned in the war. In March 1943, resistance heroes set fire to the Population Register in order to delete the data of the Jewish population. The Nazis used those data in their search for Jews to deport and kill them. However, the system was filmed in 1939 and those films were transferred to the city archives in 2000.” (F. van der Heide, personal communication, November 25, 2022)

While van der Heide maintained that records were destroyed to protect the population from death and deportation, other historians have asserted that the archives were burned in a failed attempt to obfuscate resistance member’s forged identity documents (Anne Frank House 2018). Family stories around the firebombing of Amsterdam’s city archives in 1943 are used here to illustrate the complexity of social discourse around records, archives, and the targeting of marginalized communities (Ketelaar 2020). While there is a lack of clear evidence to disambiguate the narratives, most agree that groups targeted by National Socialists (Nazis) were at high risk of death during the occupation. One could not assume that official recordkeeping processes were benign, objective or impartial toward these populations.

Tensions between official records and the recovery from past injustices have been a substantive topic of discussion in the archival literature. Gilliland and McKemmish considered the complex matrix of interactions inherent in archival practices:

“Records…are created and employed within a complex of official and other purposes and processes that involve and implicate many different institutional and human agents…*This situation is particularly problematic when one contemplates the roles that recordkeeping can play in both obstructing and promoting social justice, human rights, and recovery from past injustices and conflicts*”(Gilliland and McKemmish 2015). (Emphasis added.)

The roles of records within inquiries regarding social justice, human rights and past injustices have become central to critical paradigms of archival research, resulting in the development of discourse around foundational concepts regarding agency for the communities who are the subjects of records, such as participative archiving (Gilliland and McKemmish 2015) and archival autonomy (Evans et al. 2015):

This kind of research, which is increasingly being employed in archival studies, “moves beyond explanation to a *moral and ethical critique of the design, development, implementation and impacts” of recordkeeping and archiving practice…to overcome disadvantage, exploitation, disempowerment, domination and disenfranchisement*” (Gilliland and McKemmish 2015). (Emphasis added.)

However, existing archival literatures lacked the vocabulary to describe interactions which produced records rooted in mistrust toward the subject or hostile toward the subject’s voice. As such, this paper introduces the concept of ‘hostile recordkeeping practices’ to refer to the production of records anchored in disregard of harm toward the subject of the record, records based on an assumption of mistrust toward the subject of the record, and records created with a deliberate indifference of care for the subject of the record. This paper establishes a critique of the impact of hostile recordkeeping practices throughout the life of a record, both in terms of the responsibilities to professional ethics (Caswell and Cifor 2016; Varkey 2021) as well as in terms of the relationship between the record and the subject of the record (McKemmish 1998; Wakimoto et al. 2013; Evans et al. 2015; Caswell et al. 2016; Cifor 2016).

## Archival theory, gender theory and the social construction of records

Discourses in archival science have contributed to the understanding of records as complex, socially constructed artifacts:

“Traditional premises in archival theory and practice hold that archival records are authentic as to procedure and impartial as to creation because they are created as a means for, and as a by-product of, action, and not for the sake of posterity…The post-Positivist view of records embraces the record as a socially constructed and maintained entity” (Trace 2002).

This paper engages with tensions exposed by the conception of records as ‘socially constructed and maintained’ and argues for consideration of social interactions during the record creation process. For marginalized populations, these initial interactions often impact whether their voices, lived experiences, and realities are reflected in the records (Gilliland-Swetland 2000; Flinn et al. 2009; Wood et al. 2014). Medical records, like archival records, are usually treated as “neutral repositories of facts” (Belton 1996; Yakel 2001; Schwartz and Cook 2002). Health care providers do not typically view records in terms of being ‘socially constructed and maintained’ and many providers do not think of records as “active sites where social power is negotiated, contested, confirmed” (Schwartz and Cook 2002; Raney et al. 2021; Dozier 2022). Building from the concept of records as social artifacts, this paper approaches recordkeeping as a ubiquitous social practice with additive impacts beyond the scale of a single individual. Spade (2011) argued that recordkeeping policies created sites of active disenfranchisement for entire populations, which he termed administrative violence. Spade’s arguments also tied gender records into the production and collective maintenance of social norms:

"People who find the commonly evoked societal norms used in the classification familiar and comfortable tend to take these classification systems as neutral givens in their lives" (p. 140-41).

Spade argued that biased administrative policies and practices created “vectors of vulnerability and security” (p. 138) which reproduced systems of harm and social marginalization. Spade’s concept of administrative violence parallels a related concept of structural violence. Farmer (2004), drawing from his experiences in medical anthropology, summarized:

"Structural violence is violence exerted systematically—that is, indirectly— by everyone who belongs to a certain social order: hence the discomfort these ideas provoke in a moral economy still geared to pinning praise or blame on individual actors" (p. 307).

The concept of hostile recordkeeping draws from the idea that recordkeeping practices may create complex vectors of vulnerability and disadvantage for entire populations. In addition to drawing from concepts such as administrative violence and structural violence, hostile recordkeeping also invokes the concept of deliberate indifference of care toward the subject. The concept of deliberate indifference invokes a willful disregard of the duty to provide care, typically resulting in further harm to the subject (Carroll 2021), which has been a common problem within trans + populations’ barriers to care (Grant et al. 2011; ABC News 2012). Medical and health care contexts are explicitly tasked with the ethics of beneficence and the avoidance of harm for patients (Varkey 2021), making instances of deliberate indifference a major concern for records involved in the provision of health care.

## Population-based observational studies

Scholars currently estimate the US trans + population to be between 1–2 million persons (Deutsch 2016; Doan and Grace 2022). Providing affirming care has broad acceptance in the US medical system (AMA House of Delegates 2008; American Psychological Association 2015), but providing care has presented unique challenges resulting from complex histories of procedural frictions between medical record systems, financial systems, employment records, social norms, legal requirements, and medical practices (Currah 2008; Van Eijk 2017; Bakko and Kattari 2020). Improving the quality of health data for trans + populations has been considered essential for addressing barriers to care (Reisner et al. 2015; Deutsch 2016) and was widely assumed to be a benign and benevolent act toward rectifying systemic erasures (Bauer et al. 2009; Callahan et al. 2014; Cahill et al. 2015; Burgess et al. 2019; Lau et al. 2020). However, health records for this population occur at a juncture with a complex history of breakdowns, dysfunction, marginalization and stigma (Redcay et al. 2021; Kattari et al. 2021; Kronk et al. 2022).

Barriers to care for this population have been, by all accounts, staggering (Krehely 2009; Cruz 2014; Roberts and Fantz 2014; Callahan et al. 2014; Winter et al. 2016; Paine 2018). In Los Angeles, one group of providers summarized the severity of the problem:

"What we have learned from 15 months of direct service and close to 500 transgender patients accessing care on a regular basis—is that *transgender people face entrenched discrimination and abject denial of care within the health care system*" (Sussman et al. 2015). (Emphasis added.)

Grant et al (2011) summarized problems of access to care:

"Doctors’ offices, hospitals, and other sources of care were often unsafe spaces for study participants. *Over one-quarter of respondents (28%) reported verbal harassment in a doctor’s office, emergency room, or other medical setting* and 2% of the respondents reported being physically attacked in a doctor’s office."

"Unfortunately, our data shows that doctors’ knowledge of a patient’s transgender status *increases* the likelihood of discrimination and abuse…*up to eight percentage points* depending on the setting" *(p.6).* (Emphasis added.)

The National Coalition for LGBT Health explained:

“For many transgender people, perceived provider insensitivity and hostility produces an intense fear of disclosure of transgender status, itself a significant barrier to access to care. Based upon many anecdotal reports, *actual* provider hostility and insensitivity has resulted in deaths through delay and even refusal to provide urgently needed medical treatment” (Lewis et al. 2004). (Emphasis added.)

Barriers to care and denials of care, as described above, tend to be reported in the medical literature as a problem of discriminatory acts of individual providers (Redfern and Sinclair 2014). Many have attributed the problem to inadequate training (Rondahl 2009; Williamson 2010; Obedin-Maliver et al. 2011; Stroumsa 2014; Callahan et al. 2014), but studies have suggested that patients are more likely to encounter hostility if their gender identity is known to the providers (Grant et al. 2011; Sussman et al. 2015). Alpert et al (2023) interviewed patients and concluded, “various aspects of clinicians’ notes contradict, blame, or stigmatize patients, across multiple axes of oppression” while “certain medical customs set the stage for marginalizing, objectifying, and pathologizing transgender people.” (p. 970). The recordkeeping systems often played a central role in barriers to care, albeit one that was constantly evolving with evolving technological systems. One provider explained:

“All the pieces of demographic data for a patient must match across systems for the insurance billing process to work smoothly or even to work at all. For us, that means that we lost the most accurate patient data…sex assigned at birth was replaced… so that billing requests would move forward” (Ingraham et al. 2015).

An author of a clinical care guide explained:

"Once the carrier labels the patient as transgender or transsexual, many types of coverage may be routinely denied, where they would be covered for patients who are not identified as transgender or transsexual" (USCF Center of Excellence for Transgender Health 2011).

Critical inquiries into breakdowns of records are relevant, as they expose of the infrastructures of power, structural assumptions of what is recorded, and for whom (Star and Bowker 1999). Star (1991) explained:

“Precisely because it is so minor and yet so pervasive in my life it is a good vehicle for understanding some of the small, distributed costs and overheads associated with the ways in which individuals, organizations and standardized technologies meet” (Star 1991).

Trans + populations contend with high distributed costs associated with the intersections between their records and individuals, organizations, and standardized technologies (Grant et al. 2011; Thompson 2016; Herman et al. 2017; Van Eijk 2017; Feldman et al. 2021). Trans + populations have had a complex history with medical interactions where their voices were discounted, dismissed, or treated as inherently deceptive (James et al. 2016; Bindman et al. 2022; Fuentes et al. 2023). In the past, providers faced legal liabilities based on the legal argument that trans + persons were essentially ‘too mentally ill’ or ‘too unreliable’ to be able decide for themselves whether to have surgeries or other medical procedures. Ettner et al (2007) wrote:

"The real crux of the medicolegal drama was whether a transsexual's consent was valid. If the desire for surgery is a delusion, an obsession, or a symptom of complex psychopathology, then an informed consent lacks legal validity, therefore, the issue hinged on the etiology of gender dysphoria. Was it a 'real' condition or a psychiatric disorder?" (Ettner et al. 2007).

Trans + populations shoulder the cost of social biases around assumptions of deceit, social pathology and deception (Hale 1998; Sloop 2000; Beauchamp 2009). Depictions of trans + voices as inherently deceptive are a widespread and longstanding finding in media and cultural studies (Currah et al. 2006; Stryker 2008; Beauchamp 2011; Cifor and Rawson 2023), suggesting that bias and stigma play a substantial role in barriers to care (Bauer et al. 2009; Roberts and Fantz 2014; Redcay et al. 2021) as well as contributing to barriers to establishing fair representation in the records (Movement Advancement Project 2017; Christian 2017; Anderson et al. 2023). Social narratives are also observed to work in conjunction with a lack of functionality in recordkeeping systems to exacerbate barriers to care (Burgess et al. 2019; Lau et al. 2020; Alpert et al. 2023).

Without having experienced biased hostility in a healthcare environment firsthand, it can be difficult to grasp how deeply disruptive these experiences can be. One Los Angeles focus group participant commented:

“I feel like now with the anti-trans bills… the whole spectrum [is] being under the microscope because of political reasons and religious beliefs. I feel like *we are more of a target than we’ve ever been*. So, I, personally, just don’t go out into society unless I have to… *I literally do everything from home just to feel safe* and just to not even put myself in a position to be [under] threat, because of the way that the world is going at this kind of moment” (Fuentes et al. 2023). (Emphasis added.)

The lack of affordances to safeguard sensitive medical information leaves trans + populations as doubly vulnerable. First there is the vulnerability of facing hostility during a health care interaction. Second is the vulnerability of a record which permanently perpetuates a stigmatizing representation of the interaction. The lack of ability to safeguard oneself and one’s sensitive medical records remains visceral in the silences and anxieties articulated by members of these populations across the literature. Furthermore, the fear of loss of control of the records is widespread within trans + populations and has been documented as contributing to delaying and avoiding care (Herman et al. 2017; Mealins and Brown 2023).

## A case study of hostile recordkeeping

The original formulation of this paper involved an exploration of experiences from research participants around intentional and unintentional disappearances in the records.^1^ In sharing their narratives of work-arounds, friction and breakdowns, participants shared rich stories of successfully negotiating systems of bias and disadvantage. However, escalating politicization and outright criminalization of the provision of health care for trans + populations (Mallory and Redfield 2023) presented an unacceptable risk to professional ethics of care for protecting research participants. As Murphy noted, “once data are made publicly available, the researcher loses control over how they are used” (2021). Instead, in order to provide a foundation for the importance of the concept, the paper presents a case study which illustrates the intersections of records and hostile recordkeeping practices. In the case study, the patient’s narrative is juxtaposed against the records and supported with perspectives from the published literature. Patient narratives published in contrast with the record itself are infrequent and almost never include excerpts of actual health records (Alpert et al. 2023). Jane Fry (1974) co-authored one such account five decades ago, but since then, scholars have not been able to bring patient’s voices directly into dialog with the records on the record.

The patient, referred to here as Q [they/them], shares the story of failing to negotiate systems of friction and disadvantage. It begins their narrative with a common occurrence in LGBTIQ + lives.^2^ Q’s life was threatened in a targeted assault, multiple times over a series of months, by an unknown person or series of persons. Q explains the impact:

“Although I was successful in deterring the attack, I failed to collect evidence of being the target of a crime. There were no obvious injuries, no known witnesses, no concrete description of the attacker, and no video footage of the altercation. I had failed to document an interaction where a stranger tried to take my life. It was profoundly upsetting. I was deeply traumatized. I no longer felt safe in the world. I was unsure as to when or whether I might be targeted again, and if so, when. Due to the nature of the altercation, I had no doubt that my effeminate masculinity, my overt otherness, and my queer visibility had made me a focal point for the attackers”. (Q, personal communication, December 2023)

Without external documentation, it can be hard for law enforcement, courts or family members to take an allegation of assault seriously. Those observations aligned with Q’s experience:

“Some relatives decided I had fabricated the story while others thought that I was at fault for the attack. Looking back, I should have been more aware that some relatives might discount my experiences. My childhood had a messy history of abuse and violence, whereby certain narratives were routinely privileged to justify abusive behavior. After I came out as trans, the abusive behaviors evolved. For example, ‘George,’ cornered me one night so he could tell me how much of a mistake I was making with my life. He wanted me to know that I would “*never be a ‘real’ man*”. Little did he know that I never wanted to be a ‘real man’. *I just wanted to be myself*.” (Q, personal communication, December 2023). (Emphasis added.)

The above narrative mirrors the rejection of trans experiences of authenticity described by Bettcher (2009) and others:

“For example, an FTM who identifies as a "trans man" may find himself represented as "really a woman living as a man." One obvious feature of this denial of authenticity is that transpeople are identified in ways that are contrary to or even hostile to our own self-identifications” (p. 99).

These normative assertions of gender typically invoke a denial of authenticity and assert that a trans persons’ experiences are rooted in pathology, deceit and mental illness (Fry 1974; Hale 1998; Sloop 2000; Bettcher 2007). Q continues:

“George did not consider my experiences of trauma to be real. He concluded that my anxiety, fear, and anger could only be explained in terms of psychosis. So he decided to intervene and reported me to law enforcement as a person who was mentally ill, delusional, suicidal and actively seeking to harm others. Later, when I spoke to others in the family, they defended his fabricated statements by saying, *you were acting strangely. You needed help*” (Q, personal communication, December 2023). (Emphasis added.)

For Q’s relatives, it was unfathomable that Q might have been the target of homophobic or transphobic violence. They concluded Q must have created the problem and must therefore be endangering others. The theme of assigning a parallel narrative of deceit and pathology recurred for Q over the days which followed. Q explains how they came to believe that gender and queerness were at the heart of the problem:

Earlier that day I had dressed up for a holiday party. I had been having a hard time and wanted to cheer myself up with a silly costume. So, I aspired for something provocatively gay, if not drag-queen adjacent. I drew inspiration from Ru Paul and Richard Simmons. I had a pageboy purple wig, embarrassingly short red running shorts, a tube top, and a sporty red training jacket. When things blew up around the police report, the officer found a disheveled drag queen sitting on the curb of a clean suburban neighborhood, crying into an unkempt purple wig with dark smears of mascara running through a sprouting stubble of five o’clock shadow. It was George’s word against mine, and the validity of George’s assertions were taken for granted. There was nothing I could say (Q, personal communication, December 2023).

**Fig.1:**
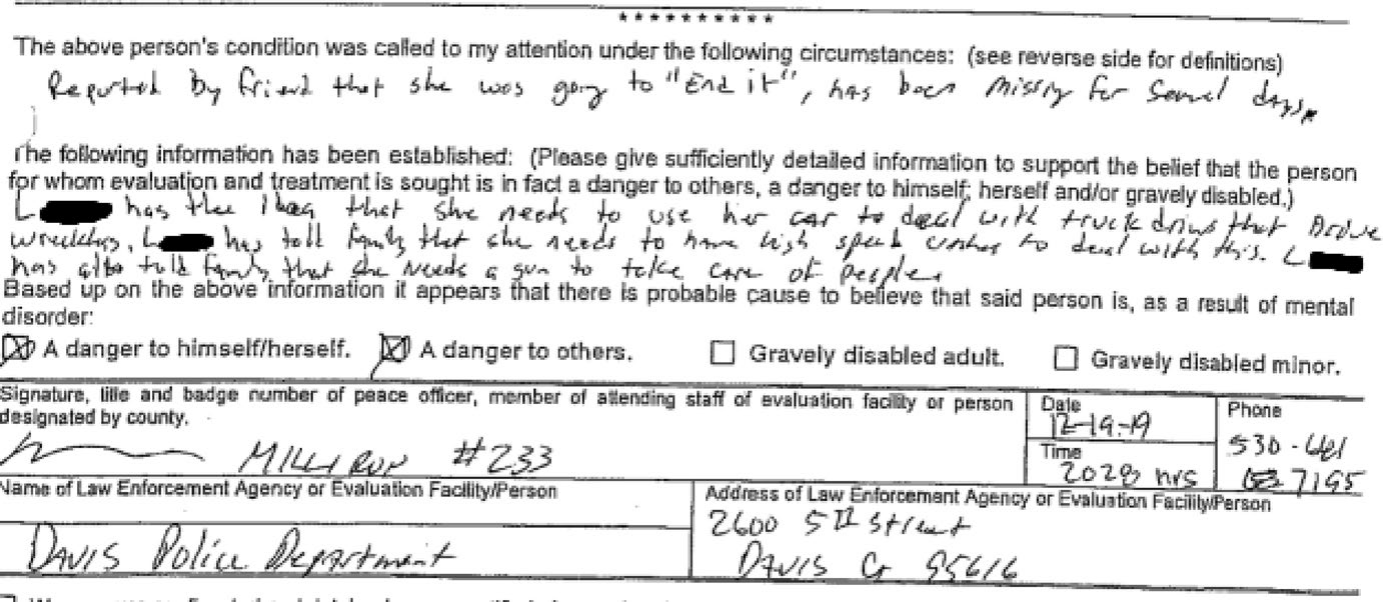
Excerpt 1 of medical record

In the resulting police report,^3^ the responding officer presented George’s fabricated narrative as established fact (Fig. 1).

The assertions presented by Officer Milliron appear to be adequately substantive, but lacked any of the information typically expected of legal documents required to detain an individual. The friend’s name is not recorded. The period during which Q was alleged to be missing is unrecorded. The legal threshold for detention depends on a person being in active and immediate danger of harming oneself or others. There is no information recorded about where alleged statements were made, on what date or to whom. At the time of the interaction with law enforcement, Q was not in possession of a vehicle and was not in possession of a firearm. Instead of providing evidence, the affidavit presents a formulation of bias in the evidence where one narrative is privileged and any evidence which might contradict it is ignored, dismissed or disregarded.

Q explains the bias in the evidence, using the example of the assertion that they had been “missing for several days”:

After all that trauma, I needed to take some time away from my family. My family had concerns about my driving, so I took the train. While at the train station, I got sick and ended up becoming unresponsive. It turned out to be a health issue, specifically a UTI and hypoglycemia. I spent a day at the hospital for observation. Normally, nothing about this type of interaction would be considered a case of a missing person and it certainly would not warrant an involuntary psychiatric detention. But the officer did not believe any of these details. The family members pushing the narrative of a mental health crisis were using the situation to evade responsibility for some $50’000 in debt I had paid on their behalf. It wasn’t about the truth. It was about making up a plausible story to justify their actions (Q, personal communication, December 2023).

The tensions in the police report reflect multiple types of bias in recordkeeping practices commonly experienced by trans + populations. The first type of bias revolves around privileging a narrative of pathological mental illness provided under questionable circumstances. The second type of bias involved the reporting officer exclusively referring to Q by the name provided by George. Q had discontinued using this name years prior. Using a previous name further demonstrates the power of exclusion and lack of voice in the record. Q explains:

The officer did not take the time to speak with me about the situation, ask me my name, or allow me to provide a statement on the information provided to him. Instead, he accepted the narrative of pathological mental illness without question, used a name which I had discontinued using years prior, and alternately misspelled both the allegations and my name in various places. It was an odd contradiction between the record, which was a legally binding report about me, and my actual voice, which was entirely excluded from the recordkeeping process and from the document itself (Q, personal communication, December 2023).

Reports of experiences such as these are ubiquitous in the literature and commonly cited within the population. Q continues:

The experience of implicit bias and denigration of trans women’s voices is widely documented in the community, which is why I understood my rumpled purple wig, smeared mascara, and unshaven beard to be directly related to my perspective being excluded from the police report (Q, personal communication, December 2023).

Without an understanding of the power of these social interactions in the production of the record, one cannot manage the records in a way that overcomes disadvantage, exploitation, disempowerment, domination and disenfranchisement as per Gilliland and McKemmish (2015). However, the police report was just the beginning. Q’s narrative goes on to show how one problematic interaction can snowball into a life-altering nightmare:

After the intervention by law enforcement, I was taken to the nearest emergency room where the officer attested that he had reason to believe I was currently “actively seeking to harm myself or others.” Based on his attestation, I was held involuntarily for a psychiatric evaluation and given heavy sedatives. After about 24 hours in limbo in the ER, I was taken to a county psychiatric hospital several hours away.

It was after midnight when I arrived and was met by the admitting nurse. We were in a small alcove with an attached restroom, full of welcoming informational pamphlets and inspirational posters. Due to a combination of heavy sedation and a long ambulance ride, I urgently needed to use the restroom. But I was restrained on an ambulance gurney. I told the nurse that I urgently needed to use the restroom. She sighed and said, *“If you can’t hold it and wet the bed, you’ll just have to lie in it until we finish the paperwork”*. I tried to answer her questions, but her words burned themselves into my psyche. I could not continue. I couldn’t stomach being tied up and forced to urinate on myself by a licensed health care provider under the color of law. For lack of a better solution, I refused to do any more paperwork until I was allowed some means by which to relieve myself (Q, personal communication, December 2023). (Emphasis added.)

**Fig. 2:**
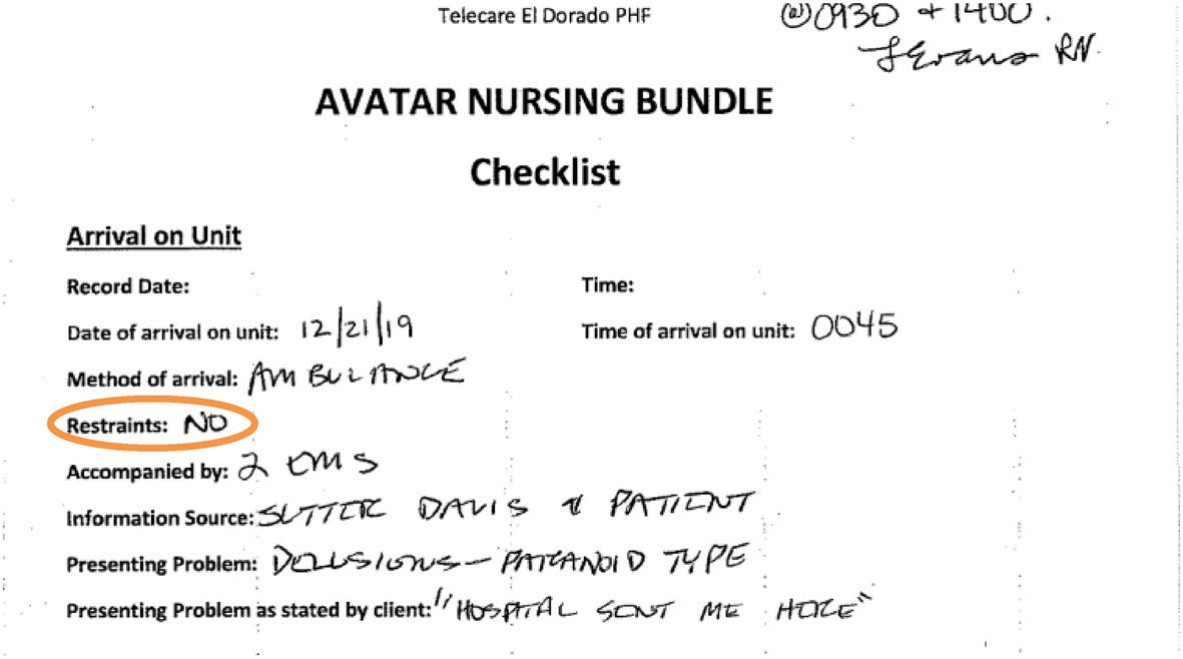
Excerpt 2 of medical record

Several years later and after multiple requests, we were able to get a copy of Q’s hospital records. Q knew the interaction was flawed, but was surprised to learn that the records had been falsified. The records indicated that no restraints were used (Fig. 2).

It is unclear why the records were falsified, but federal regulations for hospitals specify:

“All patients have the right to be free from physical or mental abuse, and corporal punishment. All patients have the right to be free from restraint or seclusion, of any form, imposed as a means of *coercion*, *discipline*, *convenience*, or retaliation by staff” (Code of Federal Regulations 2018). (Emphasis added.)

**Fig.3:**
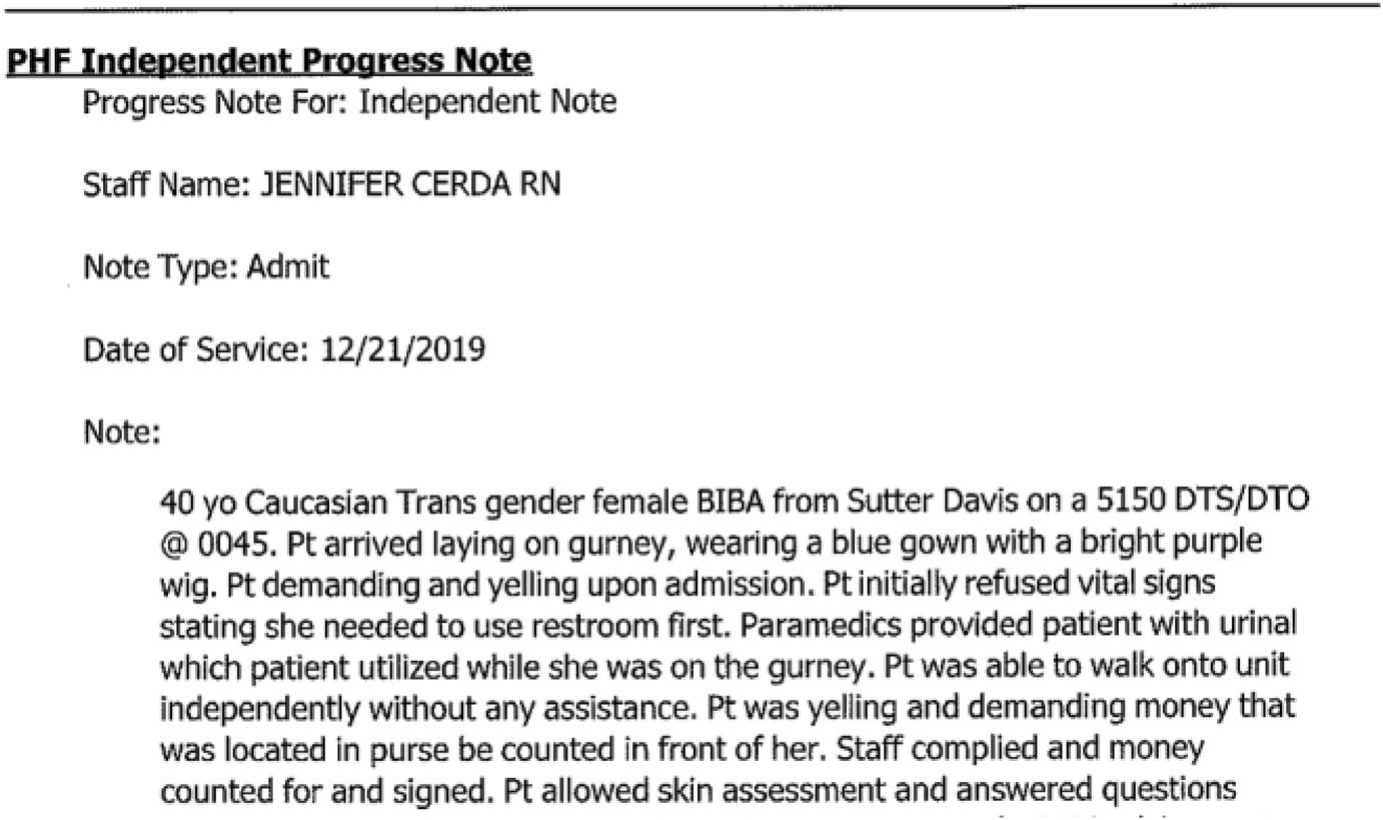
Excerpt 3 of medical record

These assertions of policy present a powerful statement in favor of the patient, but the information included in the record is controlled entirely by the staff applying the restraints. The nurse was free to portray the patient as an obstinate and demanding trans woman who insisted on urinating in front of staff (Fig. 3).

Without a narrative from the patient, a reader of the record would never have known about restraints being used to coerce the patient into compliance with the nurse’s orders. In erasing the evidence of coercion, the records suggest that the patient chose to urinate in front of staff. Q describes the experience as “unspeakably dehumanizing” for a patient in a health care setting. The patient’s narrative presents a stark contrast between the official record and the nurse’s role in the interaction, made invisible under the color of institutional authority. One could attribute the hostility in the interaction as an individual interpersonal issue. However, characterizing it as an interpersonal issue ignores the frequency of these types of experiences of trans + populations in health care contexts (Grant et al. 2011; Spade 2011; Feldman et al. 2021).

**Fig. 4:**
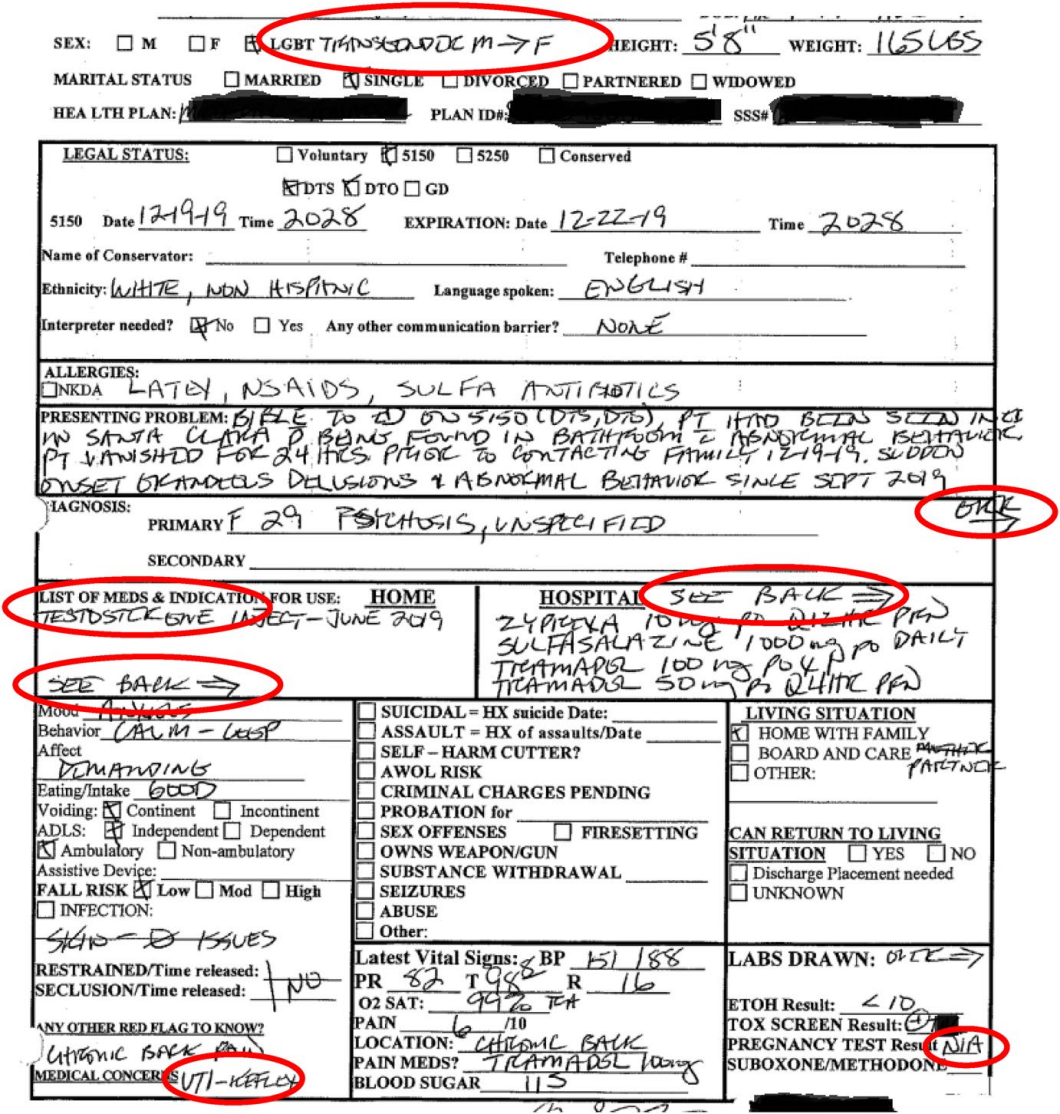
Excerpt 4 of medical record

One of the central tensions in the record is the problem of gender itself. The intake records mistakenly describe the patient as a ‘born male, transgendered female’, although the institutional scans excluded the rear side of the paper originals, leaving out much of the content of the original record (Fig. 4).

The mis-assessment of gender occurs in the initial patient description and is reaffirmed throughout by staff, even after Q begins to menstruate:

I had terrible PMS and was bleeding heavily, so I asked about some menstrual supplies from my belongings. Rather than being concerned about a potential source of abdominal bleeding or identifying an error in the record, nurses informed me that I did not have a uterus and that I did not require access to menstrual products. I resorted to stuffing paper towels down my pants with the embarrassment of a thirteen-year-old. (Q, personal communication, December 2023)

**Fig. 5:**
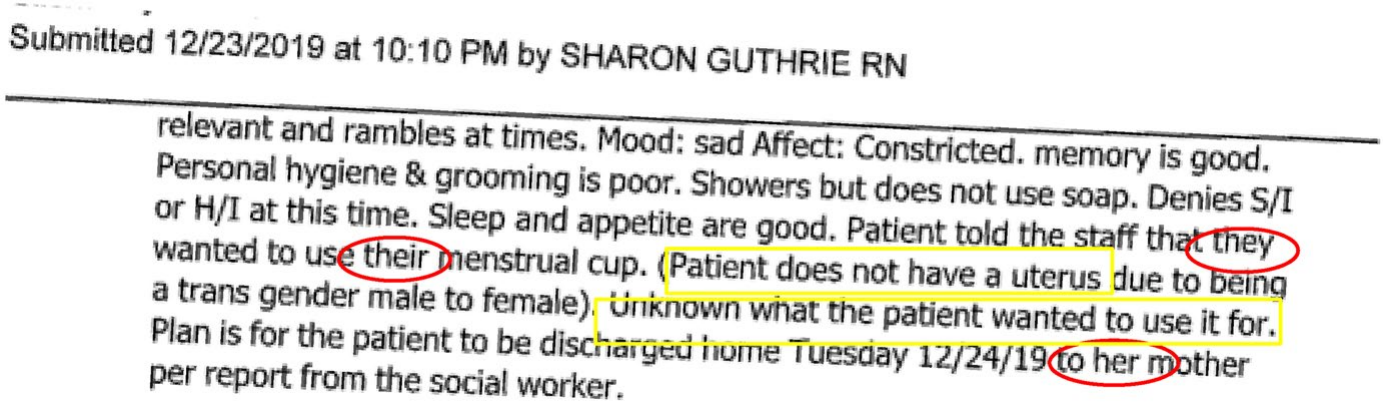
Excerpt 5 of medical record

The nurse faithfully documents the details of the interaction, including commenting about the patient’s uterus, but does not seem to want to acknowledge that the patient is bleeding (Fig. 5).

Typically, a mistake in gender designations would be considered unremarkable and a minor medical error. However, unexplained bleeding in absence of a uterus is considered a genuine medical emergency. The nurses’ refusals to acknowledge the report of vaginal bleeding can be understood as more than simple medical error, but as a willful refusal of care for the patient. In the record, the willful refusal to provide care is erased in favor of describing the patient as “bizarre” and requiring “further [involuntary] hospitalization” to address “patient’s inability to communicate effectively and engage in *behaviors within the social norms*” [emphasis added]. In looking at the situation of record production, one can see the complexity of blind spots created by bias in the recordkeeping practices. The recordkeeping practices enabled the nurses to present a one-sided devaluation of the patient’s voice and enabled the willful denial of care.

A willful denial of care and open disregard of medical symptoms were repeated themes throughout the hospitalization. Q explains:

Three days passed between when I arrived at the facility and when I received access to my prescriptions which control an inflammatory autoimmune disorder. Three days may not sound like much, but the abrupt cessation of medication resulted in immediate and debilitating physical illness. I lost my appetite, had severe pain, and struggled to walk, bath or dress myself. I was too ill to care about being misgendered or being denied menstrual supplies. It took all my strength to lie on the ground and just keep breathing. (Q, personal communication, December 2023)

In contrast, the nursing notes present a narrative where medications were provided promptly and the patient’s concerns were dismissed as psychosomatic. However, hospital records have conflicting information on which medications were provided and when. A nurse on day 3 (December 22 2:41 pm) wrote:

“Client very focused on her medications…States she is nausea [sic] and feels sick due to missing her medications. *Still waiting for our Pharmacy to deliver some*. Med Nurse has spoken with them several times today, *awaiting delivery*.”[emphasis added]

**Fig. 6:**
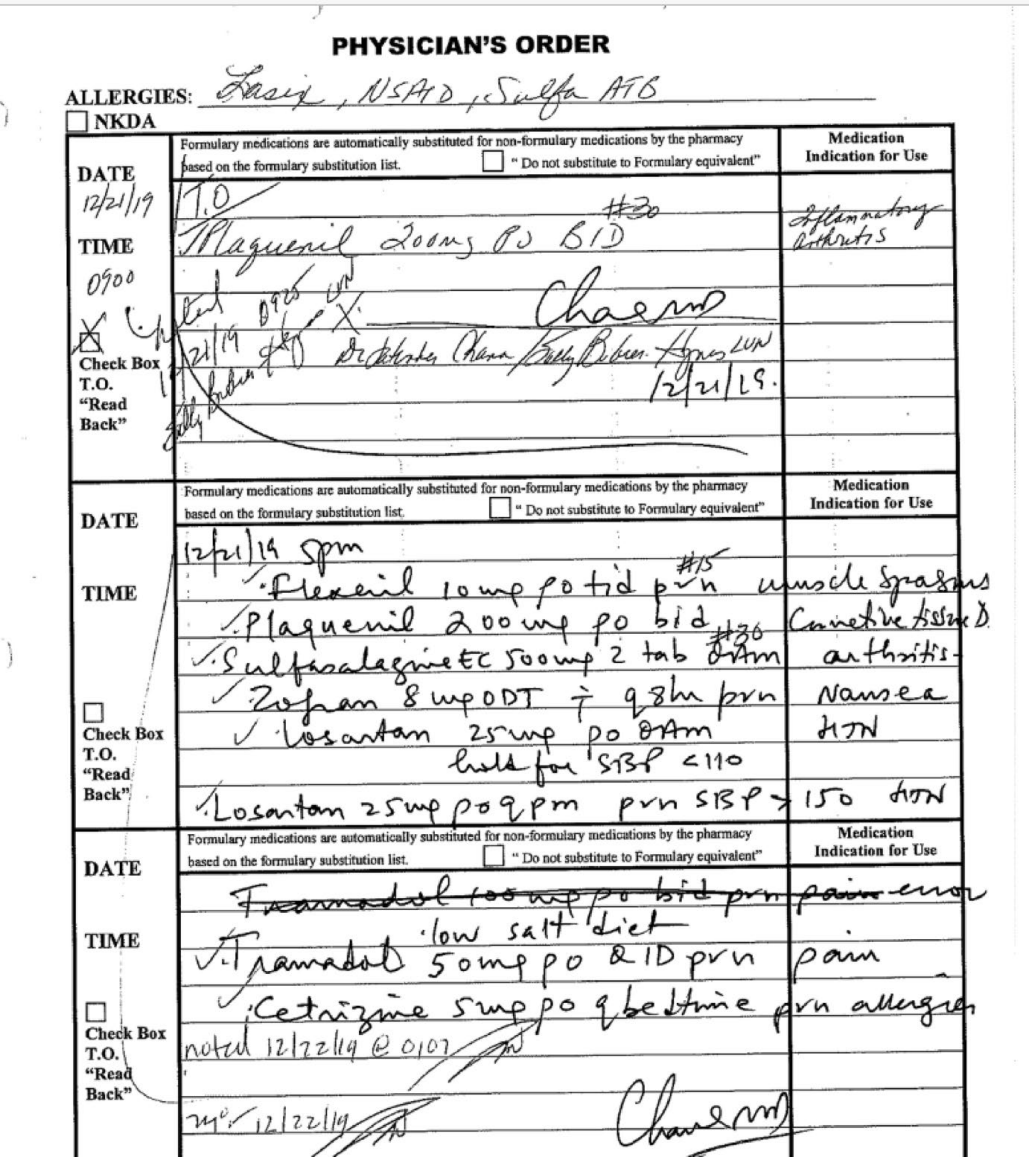
Excerpt 6 of medical record

A highly detailed reading of the record is required to find evidence that medications were missing or delayed. If the timestamps are analyzed, one can see that the attending physician failed to review the chart in a timely manner and that nursing staff failed to order the patient’s medications promptly. Below is a copy of the physician’s order for the medications which were not ‘noted’ (e.g., ordered by nursing staff) until more than 40 h had passed post-arrival at the facility, and almost three days after being detained. Hospital policies specify that these types of medications are to be ordered within 24 h of arrival (Fig. 6).

**Fig. 7:**
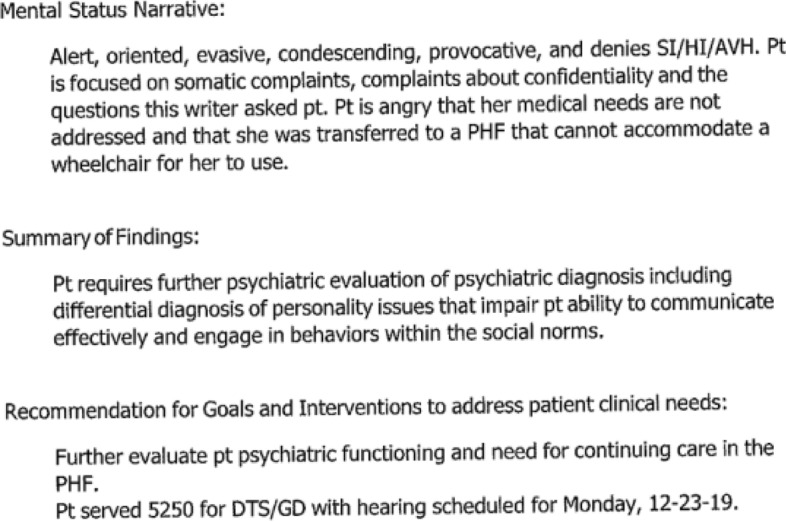
Excerpt 7 of medical record

Each symptom of physical illness is presented in the record as pathological. Q became acutely ill as a result of the lack of medical care for a known autoimmune disorder. After reporting the illness to no avail, Q finally insisted on seeing a doctor. Rather than acknowledging that medications were not provided in a timely fashion, the nurses attributed the reports of illness and requests for medical treatment as evidence of a personality disorder (Fig. 7).

At the time the above status report was written, on December 22 at 1:03 pm, Q was acutely ill with an uncontrolled auto-immune condition precipitated by the lack of access to routine medications. The patient’s physical disability and related medical symptoms were dismissed as if they were an obsessive psychological fixation rather than an acute medical situation. In a ‘Summary of Findings’ the nurse concluded, “[Patient] is fixated on medical issues, and complaints about her care here and somatic medications [sic].” Later she wrote up the patient’s repeated requests for auto-immune medications as a probable learning disability, describing the patient’s reports of illness as “beliefs about personal health” and labeling these ‘beliefs’ a “barrier to learning.” These types of interactions expose the power of the record, the potential for bias and the impact of recordkeeping practices. Nurses repeatedly neglected basic medical care in favor of biased records focused on a marginalizing narrative of pathology, delusions and psychosis. Their records create an alternate reality where all patient complaints may be disregarded as the product of delusions or as artifacts of psychosis.

If one did not speak with Q, one would believe that there were no problems with medications, no issues regarding bias and no denial of care for physical illness. Eventually, Q was able to get access to their medications and gained some traction with one of the psychiatrists:

Four days after being detained, I saw a second psychiatrist who took the time to listen. We discussed my preferred name, pronouns, and he evaluated the presentation of psychiatric symptoms. He did not find evidence of delusions, suicidal ideation or evidence of harming others. He revised the psychiatric diagnosis from psychosis to adjustment disorder, citing stress in my personal life as a contributing factor (Q, personal communication, December 2023).

**Fig. 8:**
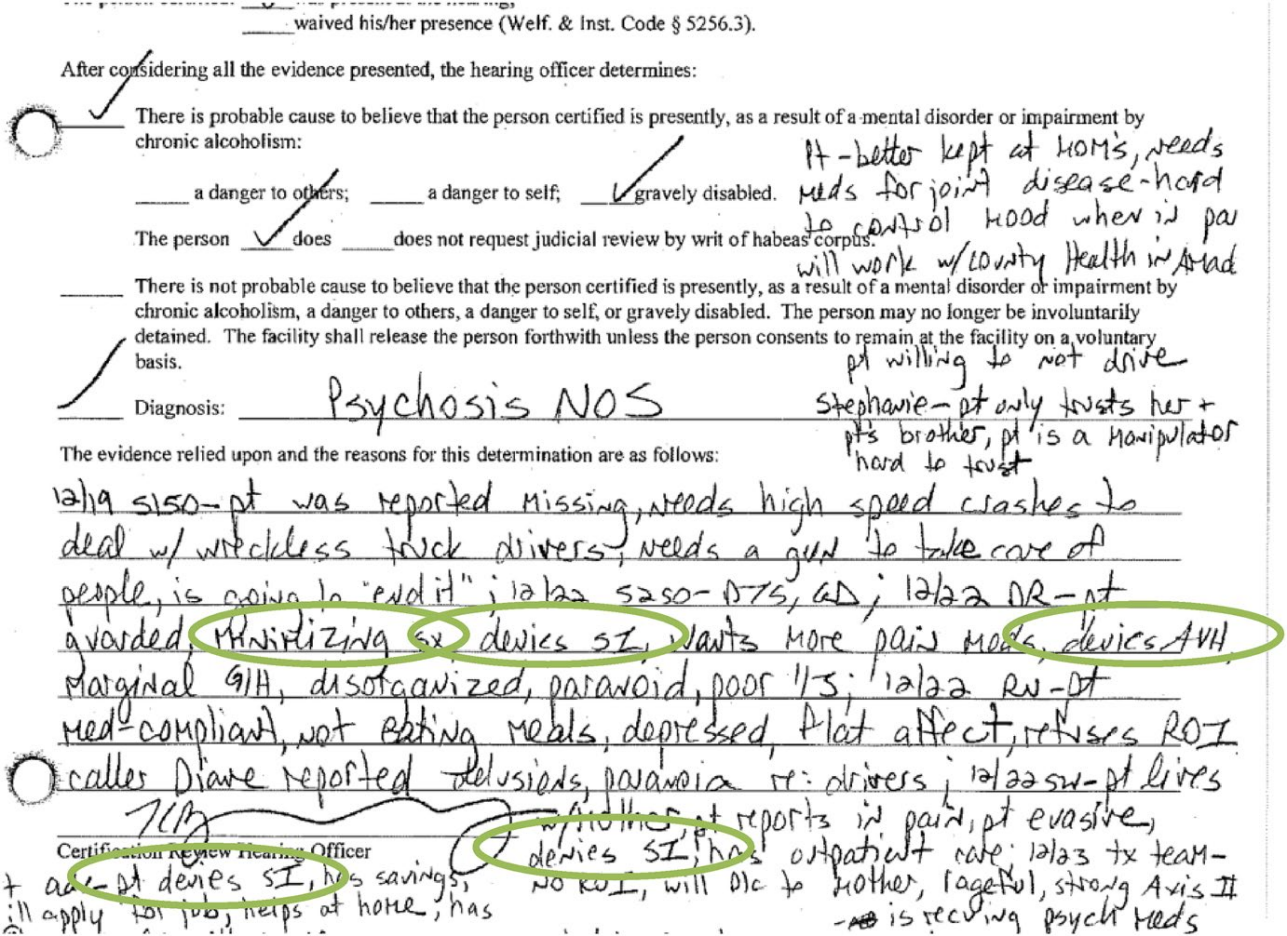
Excerpt 8 of medical record

However, the hearing officer chose to ignore the psychiatrist’s clinical evaluation in favor of the other, more biased records (Fig. 8).

The sections which are circled show moments where the patient’s voice is included but repackaged as pathological manifestation of a psychiatric disorder: “guarded,” “minimizing symptoms,” “denies suicidal ideation,” “denies auditory/visual hallucinations,” “denies suicidal ideation,” “evasive,” “denies suicidal ideation.” The question of whose voice was represented, in whose terms, under what kind of lenses and for what purpose are key features which demonstrate the document’s complexity as a socially constructed artifact.

**Fig. 9:**
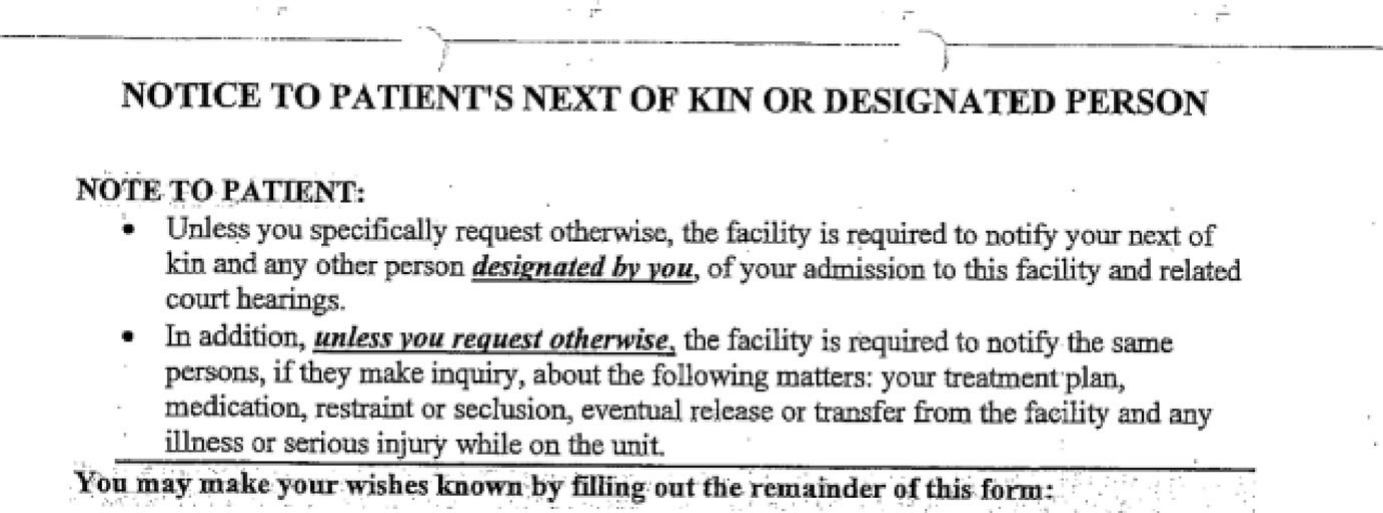
Excerpt 9 of medical record

Recordkeeping practices such as these contribute to devaluing and negating the patient’s archival autonomy. In the records, the lack of respect for the patient’s autonomy is most visible in the conversations about giving permission to release information. Because of a history of abusive behavior in their family, Q refused to designate relatives on the hospital’s ‘release of information’ (ROI) form (Fig. 9).

**Fig. 10:**
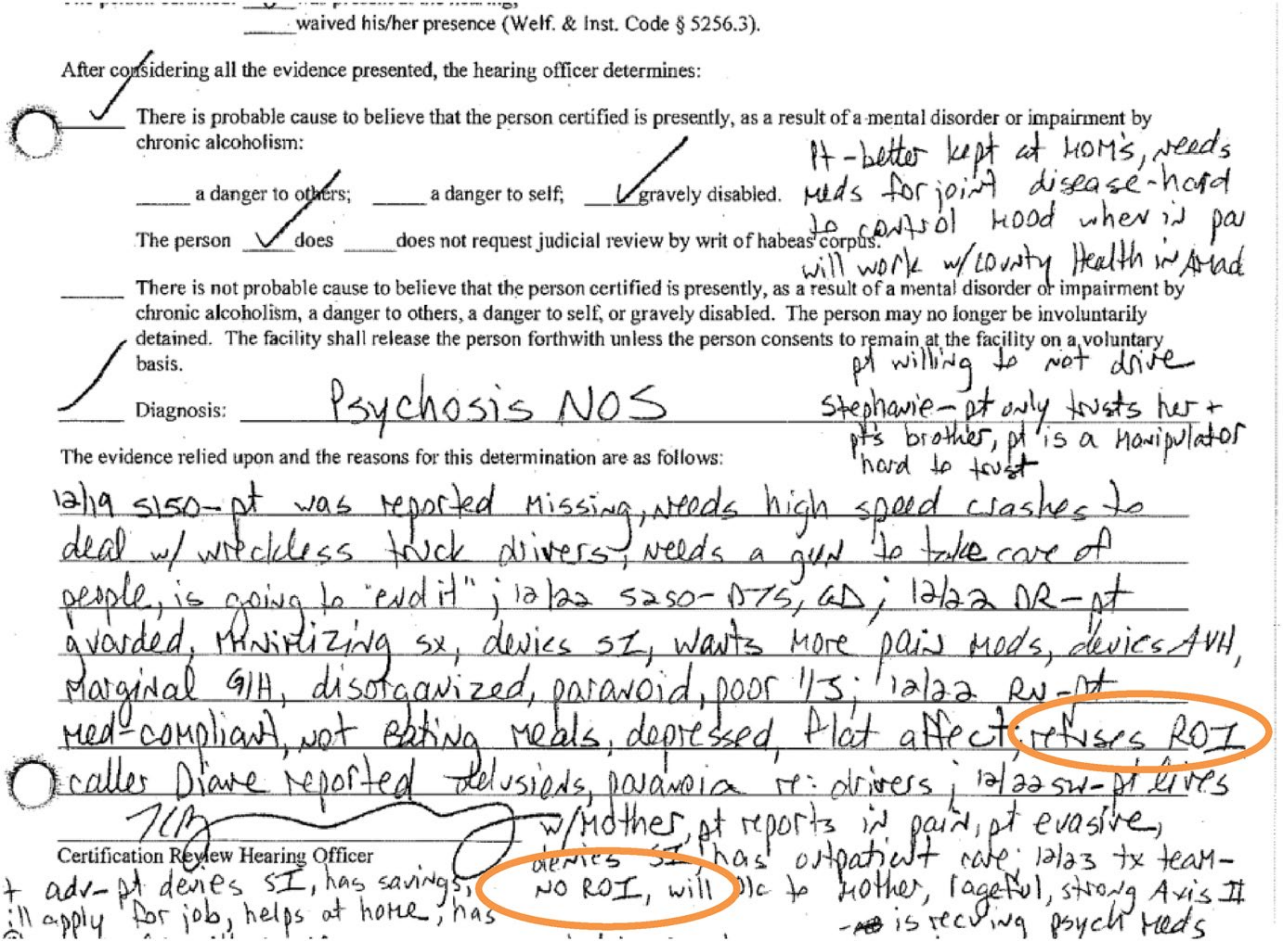
Excerpt 10 of medical record

A refusal to release medical information to third parties is a legally protected act for individuals in California. However, the hospital would not accept the patient’s designation as legitimate. The designation was cited multiple times as evidence of a mental health disorder in provider notes and within the document certifying the findings for the State (Fig. 10).

The inclusion of the decision to release of information as evidence of mental illness is in disregard of the rights of patients to make decisions around the sharing of medical information. The repeated references to the patient’s decision about the release of information (ROI) exemplify the erasure of archival agency and denial of first-person authority by staff. The institutional endorsement of these actions via court proceedings demonstrated an explicit disregard of the patient’s rights to determine access to their records. This disregard of patient agency over the records was reiterated when Q requested for the institution to remove or correct the erroneous information from their record. The institution refused to remove the stigmatizing language and refused to correct the errors around gender markers. Q’s case demonstrates profound tensions around medical records and stigmatizing language. The records are presented as “authentic as to procedure and impartial as to creation because they are created as a means for, and as a by-product of, action” (Trace 2002). Instead, the records perpetuated untrue, biased and unjust narratives about the patient.

## Discussion: reflecting on the record

The patient narrative and associated medical records provide an entry point for researchers to engage with the complexity of health records around questions of provenance, validity, safety, invisibility, hostility, and voice. The misrepresentation of the patient was so substantial that the provision of psychiatric evaluation could not be assessed from the records. Was the patient a post-op trans woman? Or a transmasculine female? Did the patient have a uterus? Was menstrual bleeding a pathological delusion? Did the patient have a physical disability? The misrepresentation of the patient was based in a disregard of the authenticity of the individual. In doing so, staff authored a new narrative of an angry trans woman who necessitated involuntary detention in a locked psychiatric facility in order to teach her “*to communicate effectively and engage in behaviors within the social norms*.” (see Fig. 7, emphasis added.)

In most regards, the record was misleading and misrepresented basic facts about the individual. The patient’s biological sex, birth gender, and gender identity were misidentified and misattributed. The patient’s name was misspelled in multiple instances, including in certification documents sent to the State Department of Justice. Medication logs were misleading and incomplete. There were conflicting reports of psychiatric diagnoses, conflicting statements about whether the patient lived alone or resided with family, and conflicting findings around the risk of harm. The hospital record even includes pages from another (unidentified) patient.

Despite the ease with which providers can create biased, stigmatizing, or error-ridden records, health and government records systems lack effective mechanisms to address bias. Patients are denied the authority to request the removal records for instances when they have been misdiagnosed, discriminated against, or been the subject of falsified statements provided to law enforcement. Even with the obviousness of the errors in Q’s case, there was no functioning mechanism to expunge the erroneous records. El Dorado Telecare has refused all requests to correct errors in the records. Instead, they deferred responsibility to staff members who have ‘since left the facility.’ While staff members may have left the facility, the impact of the records remains and casts a long shadow. These records were created with the explicit purpose of legally revoking the patient’s individual freedoms and personal autonomy. The records remain on file as court documents deposited with the State’s Department of Justice.

While the above recordkeeping interactions could be viewed as isolated, interpersonal problems, doing so fails to address the widespread reports of biased and stigmatizing interactions from individuals within marginalized populations. Ethical questions around how to approach and document evidence of hostility and bias in the recordkeeping environment are critically important for researchers engaged with questions of how records are created, retained and used. Interactions in healthcare contexts may be individually hostile or productive, but the lack of ability for the records to be corrected, contested, or expunged produces an environment where damaging records may be proliferated downstream across multiple systems without regard to bias, human error, stigma, marginalization, or quality control.

## Conclusion

Medical records, legal certifications and identity documents have enormous power in their positivistic portrayal of truth. Acts of confirmation bias, acts of erasure, and exclusions of marginalized voices are invisible in the final records, as if truth required no such documentation of evidence. Moreover, trans + populations often bear the burden of negative outcomes resulting from bias, friction, and errors in recordkeeping processes. The potential harm of hostile recordkeeping extends well beyond the scope of an immediate hospital stay, with the potential to affect access to care, access to identity documents, to be used in legal proceedings, and to result in criminal proceedings. As health records become increasingly portable between states, contexts and health systems, we are obligated to ask deeper questions about safeguarding marginalized populations from hostile future uses. Trans + populations have recently seen dramatic increases in healthcare politicization in the US, including criminalizing and/or legislating the exclusion of care. US records systems lack the ability to protect sensitive information recorded about LGBTQ + specific care or reproductive health care. For a population which has regularly sustained failures of care and recordkeeping, the lack of agency in health and government records presents one of the largest barriers to equity and transformative justice for this population.

As a discipline, we must also ask deeper questions about learning how to identify indicators of hostile recordkeeping. While many community providers were highly sensitive to the disparities and distortions created by recordkeeping practices, recordkeeping systems currently lack any mechanisms to identify or address bias in the records. Future archival and medical discourses alike need to engage with the situation(s) of creation, the acts of recording, and, most importantly, personal agency in the negotiations of allowable uses for the records for trans + populations. Future inquiries would benefit directly from the development of a critical analytical lens to understand and articulate observations of hostile recordkeeping environments, indicators of bias, and mechanisms for surfacing issues of trauma, safety, stigma, and repair. Much work is needed to safeguard these populations and provide recordkeeping environments which conform to an ethics of care for this population.

## Footnotes

1 During the research process, I requested permission to archive, identify and make the interviews openly available as an oral history project. Included participants provided full permission to archive and make available their contributions.

2 This narrative is entirely based on real events and persons. Some details have been omitted and some simplified due to the limitations of space.

3 The Davis Police Department refused to provide the patient with a copy of the resulting police report. However, a copy had been provided to the hospital and had been included in the medical record for the patient.
